# A visual question answering method based on task decomposition

**DOI:** 10.1371/journal.pone.0336623

**Published:** 2025-11-13

**Authors:** Yao Cong, Hongwei Mo

**Affiliations:** College of Intelligent Systems Science and Engineering, Harbin Engineering University, Harbin, Heilongjiang, China; Tongji University, CHINA

## Abstract

Visual question answering (VQA) as an interdisciplinary task of computer vision and natural language processing, estimating the model’s visual reasoning ability, which requires the integration of image information extraction technology and natural language understanding technology. The testing on professional benchmark which controls the potential bias states that the VQA method based on task decomposition is a promising approach, offering advantages in interpretability at program execution stage and reducing data bias dependencies, compared with traditional VQA methods that only rely on multimodal fusion. The VQA method based on task decomposition decomposes the task by parsing natural language and it usually parses the language with sequence-to-sequence networks. It has limitations when faced with flexible and varied natural language, making it difficult to accurately decompose the task. To address this issue, we propose a Graph-to-Sequence Task Decomposition Network (Graph2Seq-TDN), which uses semantic structural information from natural language to guide the task decomposition process and improve parsing accuracy, additionally, in terms of reasoning execution, in addition to the original symbolic reasoning execution, we propose a reasoning executor to enhance execution performance. We conducted validation on four datasets: CLEVR, CLEVR-Human, CLEVR-CoGenT and GQA. The experimental results showed that our model outperformed the comparative model in terms of answering accuracy, program accuracy, and training costs under the same accuracy.

## Introduction

**Fig 1:**
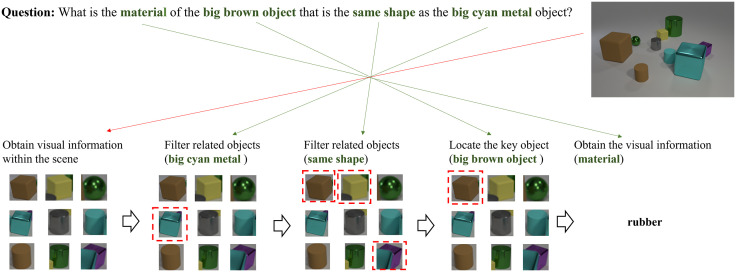
Natural language guided visual reasoning in visual question answering.

Visual question answering (VQA) is a challenging interdisciplinary task that requires a system to answer natural language questions related to a given image using the information obtained from the image [1], [2]. For instance, for the question “What is the material of the big brown object that is the same shape as the big cyan metal object?” the system needs to locate the big cyan metal object based on the visual clues provided by the image (size, color, material), obtain new visual information (shape), and then find the big brown object before finally querying its material property, as shown in Fig 1. This is a process that utilizes natural language to guide visual reasoning and is therefore considered as an important means of studying visual reasoning (which is regarded as a North Stars problem in computer vision and a key aspect of embodied intelligence [3]).

The origin of question answering task can be traced back to the Turing test. The VQA task was first proposed by Malinowski in 2014 and has since then attracted a lot of academic attention, especially after the relevant dataset was proposed (DAQUAR [4], COCO-QA [5], VQA v2.0 [6], etc.). Early VQA models mainly relied on multimodal feature fusion, where image features and text features were combined to form new features, which were then classified to obtain the answer. However, such methods, as the earliest task solutions, had limited accuracy, especially considering that there was a lot of irrelevant information during the process of fusing image and text features. Therefore, attention mechanisms were introduced to restrict the fusion of image and text features, thus improving the accuracy of answer generation. Nevertheless, the above methods do not provide interpretability during the question-answering process and we cannot know how the model obtained the final answer, it means that they may rely heavily on dataset bias, i.e., the models may not have made reasoning, but only remembered the answer through some implicit clues. The entire process is based on selecting the candidate answer with the highest probability, which lacks interpretability.

As a result, scholars have proposed a new VQA method based on modular neural network, which is based on a task decomposition framework that parsing the overall natural language question into the combination of multiple sub-tasks. By completing these tasks, the final answer can be obtained and satisfy the logical and interpretable needs of reasoning tasks. In addition, the emergence of large language models (LLM) such as GPT [7] and PlaM-E [8] in recent years indicates that general artificial intelligence guided by natural language is very likely to be realized. For robots, in particular, how to interact with the environment through natural language on the basis of semantic understanding is the key to realizing embodied intelligence. The current popular paradigm is to decompose embodied tasks through natural language parsing, and then call on low-level skill combinations of robots to complete them [9–11]. The VQA method based on task decomposition framework, which completes the entire reasoning task by parsing the reasoning steps through natural language and calling functional components to complete sub-tasks, is a miniature representation of embodied intelligence. Therefore, the research on the VQA method based on task decomposition can serve as a foundation for the study of embodied intelligence in robots. Investigating task decomposition in visual question answering enables extending these principles to robot task planning, thus enhancing robotic environmental interaction capabilities.

The most crucial issue for the task decomposition method is how to accurately decompose tasks and effectively execute reasoning based on the results of task decomposition. The task decomposition means to determine the visual reasoning goals. Previous VQA methods based on task decomposition mainly relied on Sequence-to-Sequence (Seq2Seq) networks based on RNN, LSTM etc. to parse natural language questions, and the parsing process depended on a large number of data labels or expensive pre-training costs (after the emergence of language pre-trained models like BERT). Some of these methods could adapt to standardized generated language questions, but they were limited in responding to humans’ flexible and varied natural language questions. The other methods can generate diverse languages after extensive data training, while it is difficult to generate reasonable task decomposition for multi-hop reasoning. These factors can all lead to significant drops in their effectiveness. After accurately decomposing questions in visual question answering (VQA) tasks into sub-tasks, effectively executing reasoning operations according to the decomposed steps is crucial. Most task decomposition frameworks for visual reasoning employ Symbolism to design reasoning executors. Symbolism abstracts human cognition into computable symbolic systems and achieves intelligence through rule deduction. While this approach enhances the interpretability of reasoning, its reliance on manually encoded rules limits flexibility and adaptability in complex, uncertain scenarios. Consequently, such models often remain confined to simulated environments and struggle to generalize to real-world applications.

To solve these problems, this work proposes a Graph-to-Sequence Task Decomposition Network (Graph2Seq-TDN) that accurately decomposes natural language questions into sub-tasks and performs visual reasoning according to the decomposition steps to complete visual question answering tasks. In summary, the contributions of this work are as follows: 1) We propose a Graph2Seq Task Decomposition Network, which utilizes Graph Convolutional Network (GCN) with attention mechanisms to decompose tasks; 2) The method based on pre-defined graphs was used to topologically process natural language questions, introducing prior knowledge into the graph and the heterogeneous graph message passing and aggregation methods were provided to leverage structural information; 3) The method based on adaptive graphs was also applied to topologically process natural language questions, thus reducing preprocessing costs by automatically learning graph structures through similarity; 4) On the basis of the original symbolic reasoning execution, we also propose a reasoning executor to improve reasoning execution performance in complex real world scene.

## Related work

**Fig 2:**
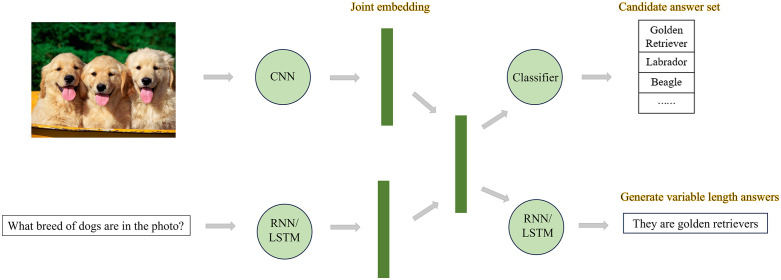
Framework based on modal fusion method.

The VQA task was first proposed by Malinowski et al., they subsequently proposed a classic VQA model based on multimodal fusion – the Neural-Image-QA model to tackle the task [12,13]. They modeled the VQA problem as generating problem, extracted image and problem features and stitched them as input, then sent it into the LSTM network to output answers. The model based on multimodal fusion takes visual and textual features as inputs, maps the two types of features to the same feature space, and then fuses these features through a simple fusion mechanism. The fused features are then fed into a linear classifier or neural network, as shown in Fig 2, the key of such multimodal fusion method lies in how to fuse visual and textual features, and a large number of scholars have focused their research on related studies. Typical multimodal feature fusion methods mainly combine visual features and textual features through affine transformation, element-wise product, outer product of vectors, matrix decomposition, etc., such as VIS+BILSTM (Ren et al. [5]), MCB (Multimodal compact bilinear, Fukui et al. [14]) pooling, MFB (Multimodal Factorized Bilinear, Yu et al. [15]) pooling, MUTAN (Cadene et al. [16]), etc.

As a traditional VQA method, the VQA model based on multimodal fusion has achieved a certain degree of effectiveness. However, relying solely on feature joint embedding is not sufficient to capture and model all feature information. The process of modal fusion does not involve understanding the question or reasoning about the content of the image. At the same time, due to the fact that a large part of the information in visual and textual features is not helpful for solving tasks, directly embedding the two features together will affect the final classification or answer generation due to irrelevant information.

To enhance question-answering accuracy, researchers prioritize improving the utilization of effective information in visual and textual data. By employing attention mechanisms, they focus on image regions relevant to questions or key phrases in questions. This approach simulates the human cognitive pattern of allocating limited attentional resources to critical aspects based on actual demands, thereby significantly strengthening neural networks’ comprehension capabilities. For example, Zhu et al. [17] combined attention methods with LSTM network. At the encoding stage of this method, the image is taken as the first input token, the image feature is taken together with text feature as input, then the attention map is output for each step, and the attention map is multiplied with visual features to generate new visual features. Shih et al. [18] directly multiplied visual features with text features to obtain attention weights, with the size of weights representing the importance of the regions. The improvement effect of attention mechanism on multimodal fusion models lies in aligning image information with problem information. Therefore, more changes have been made to the attention mechanism aimed at improving the alignment effect, typical examples include co-attention (Lu et al. [19]), intra modal and inter modal attention (Gao et al. [20]), multi-level attention (Yu et al. [21]) etc. Graph Neural Networks (GNNs) are neural networks that capture dependencies between objects by treating them as nodes in a graph, and have been widely adopted in multimodal tasks in recent years. Wu et al. [22] leverage Graph Neural Network (GNN) to learn representational relationships between semantic objects and key regions, it utilizes keywords from questions as the foundation, exploits precise regional information from images to provide cross-modal visual-textual features, and predicts answers accordingly. Yusuf et al. [23] proposed a method to enhance neighboring image region features and learn question-aware visual representations. They construct a region graph from images and employ Graph Convolutional Networks (GCNs) to integrate contextual information for enriching each region’s features. Additionally, a question-aware dual attention mechanism refines regional features at both region and attribute levels, ensuring the model focuses on critical regions that essential for answering questions. Wang et al. [24] introduced a multimodal GNN module into VQA models. On well-structured multimodal semantic graphs, this module derives unified knowledge representations from both unstructured and structured multimodal knowledge via explicit interactions. While GNN-based VQA models typically achieve local fusion of object-text relationships through structured message passing and perform reasoning via graph representations. Overall, they rely on predefined graph structures, with the emergence of Transformer and its maturation in natural language processing, researchers increasingly extend its application to visual domains. Benefiting from its dynamic adaptive properties, Transformer effectively models inter-regional relationships. Zhu et al. [25] designed a Weight-Sharing Hybrid Attention Network based on a lightweight Transformer architecture, where both visual and linguistic encoders adopt attention models without convolutional or regional feature structures. Bazi et al. [26] employed a Transformer-based Contrastive Language-Image Pretraining (CLIP) network for remote sensing image VQA tasks. This framework embeds image patches and question words into sequences of visual and textual representations, then captures intrinsic intra- and inter-modal dependencies through learned attention mechanisms.

**Fig 3:**
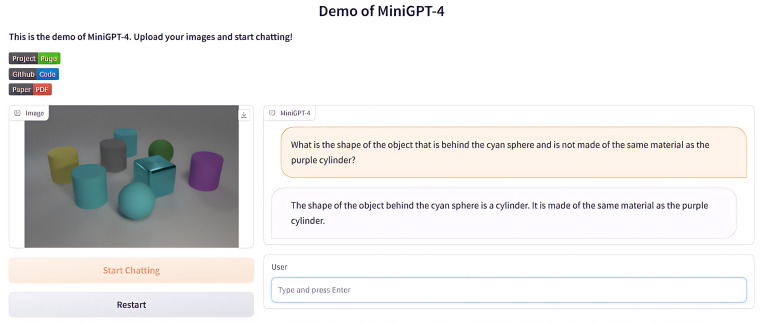
MiniGPT-4 visual answering result.

Nowadays, vision-language pre-training models based on self-attention architectures, such as ALBEF [27], BLIP [28], InstructBLIP [29], etc. become more popular and these multimodal fusion large models have achieved remarkable results in the VQA field. Moreover, with the rise of ChatGPT, LLM has attracted widespread attention. These models were trained on a large amount of data through prompt learning and reinforcement learning from human feedback, some of vision-language models was developed based on LLM’s capacity of facilitating natural language communication that can leverage their distinct and complementary capabilities, such as MiniGPT-4 [30], Cola [31] etc. However, both LLM and multimodal fusion large models still have limitations in its visual reasoning ability, especially when facing multi-step reasoning problems. The testing performance of MiniGPT-4 for VQA tasks is shown in Fig 3. The answer given by MiniGPT-4 to the question “What is the shape of the object that is behind the cyan sphere and is not made of the same material as the purple cylinder?” is “cylinder”, because it has the same material as the purple cylinder, while the question clearly requires “not made of the same material as the purple cylinder”. It is obvious that it has insufficient understanding of the constraints of the problem and does not conform to the logic of human answering questions.

The ultimate goal of visual reasoning in visual question answering tasks is to resolve cognitive-level challenges. We would prefer the machine to answer complex questions based on human logic, which means that we expect models not only to achieve correct predictions but also to provide explicit and valid reasoning paths. However, most existing research relies on implicit reasoning processes, which fail to generate traceable reasoning paths. Andreas was inspired by the fact that humans divide questions into several steps when answering them, and serialize the reasoning process of visual question answering models. The NMN [32,33] (Neural Module Networks) model constructs multiple sub-modules required for answering questions, such as “find”, “transform”, “combine”, “describe”, “measure”, etc. Afterwards, according to the structure of the problem, the combination of sub-modules is automatically generated for collaborative learning. In 2016, Johnson et al. [34] introduced Composite Language and Elementary Visual Reasoning diagnostic (CLEVR) dataset, a new VQA dataset that carefully controls the potential bias and tests a range of visual reasoning abilities. Research has shown that many VQA methods based on modal fusion exhibit a significant decrease in performance on CLEVR dataset. In 2017, Johnson et al. [35] proposed a visual reasoning model for the CLEVR dataset. They argued that to successfully perform complex reasoning tasks, it might be necessary to explicitly incorporate compositional reasoning in the model structure. Due to the simple scene of the dataset, attention would only be paid on reasoning itself. As a result, researches on this composite dataset and VQA method based on task decomposition have grown rapidly and mainly focus on NMN family. For instance, Hu et al. [36] proposed an end-to-end modular network (N2NMN) that can directly predict the module combination and layout of a problem without the help of a parser. However, both NMN and N2NMN require strong supervised information to pretrain or supervise layout strategies to obtain correct module layout and maintain good performance. If these supervised signals are lost, the model will experience significant performance degradation or inability to converge. Based on this, Hu et al. [37] further proposed a Stack-NMN method that can automatically guide the required sub-task decomposition for combinatorial reasoning without relying on strong supervised signals. At the same time, it can also ensure that the layout strategy of the module is differentiable, allowing for optimization using gradient descent method. Yamada et al. [38] proposed Transformer Module Network (TMN), which is a kind of NMN based on compositions of Transformer modules, combining the strengths of Transformers and NMNs in order to improve the systematic generalization capabilities of learning machines.

Yi et al. [39] proposed Neural-Symbolic VQA (NS-VQA), which is a typical model composed of scene parser, problem parser, and program executors. Symbolic program execution offers full transparency to the reasoning process, making it able to interpret and diagnose each execution step. Compared with NMN, NS-VQA mainly simplifies the execution of sub-tasks. Eiter et al. [40] presented a neuro-symbolic VQA pipeline for CLEVR, which relies on answer-set programming (ASP) to infer the right answer given the neural network output and a confidence threshold. The distinguishing feature of the pipeline is to fix the threshold based on the mean and the standard deviation of prediction scores, thus restricting non-determinism of object detection prediction. Meanwhile, it offers a simple yet expressive modelling language and efficient solver technology. Bao et al. [41] proposed a Confidence-Based Neural-Symbolic (CBNS) approach, which provides confidence estimation for deep learning models within neuro-symbolic systems. This mechanism simplifies the error analysis process in current neuro-symbolic VQA models while enhancing their transparency and interactivity. Johnston et al. [42] introduced a novel neuro-symbolic architecture that abstracts bottom-up information between neural networks and symbolic reasoning via Gaussian Mixture Model (GMM) probability learning. Combined with an explicit core knowledge amendment mechanism, this framework enables natural translation between natural language questions and knowledge graph queries, thereby facilitating interpretable visual question answering. Gao et al. [43] developed a hybrid neuro-symbolic reasoning model that integrates deep neural network visual and linguistic features with a symbolic reasoner connected to knowledge bases. The symbolic reasoner effectively combines visual and linguistic information with ontological relationships and commonsense reasoning to resolve complex logical questions. Additionally, it replaces traditional multimodal fusion layers in VQA deep neural networks with an innovative logical reasoning component, generating plausible answers alongside explicit logical reasoning chains.

In Table 1, we summarize the characteristics of the aforementioned visual question answering (VQA) models. It can be observed that models based on modality fusion often exhibit a simple structure (typically end-to-end architecture), are susceptible to language bias, and offer limited interpretability. In contrast, VQA models leveraging task decomposition mechanisms generate intermediate representations or sub-goals, enabling deeper logical reasoning and causal analysis. These models demonstrate stronger capabilities in complex reasoning and resistance to interference. Moreover, since the reasoning process is decomposed into multiple interpretable intermediate steps, researchers and users can intuitively trace the decision path of the model and identify whether errors occur at the perceptual or reasoning stage. This significantly enhances interpretability and controllability, which not only facilitates debugging and optimization but also bolsters user trust in the model.

**Table 1:** Comparison and summary of visual question answering model characteristics.

Method	Literature	Characteristic
**Simple feature fusion model**	[12], [5,13,14,16]	Simple structure, efficient training, low computational resource requirements, suitable for simple question answering, ignoring fine-grained interactions, susceptible to language bias, and difficult to handle complex reasoning.
**Attention mechanism model**	[17], [18–21]	Enhance interpretability, visualize attention areas, and significantly improve the accuracy of complex problems.
**Graph Neural Network Model**	[22], [23,24]	Explicitly modeling object relationships, combined with knowledge graphs, can handle open domain problems with high computational complexity and poor real-time performance.
**Transformer model**	[25], [26–29]	End-to-end training, supports long-distance dependency modeling, pre trained models have strong generalization ability, and are suitable for open question answering.
**Task Decomposition Framework Model**	[32], [33,35–43]	High interpretability, transparent reasoning process, reduced data bias, improved robustness, module design relies on manual prior knowledge, low flexibility.

For models based on task decomposition mechanisms, the most critical initial step in addressing VQA tasks is to derive reasoning objectives—that is, sub-task decomposition. Sub-task decomposition is the prerequisite for everything. Currently, VQA methods based on the task decomposition mainly validate on synthesized datasets (including images and questions). The synthesized questions are often more standardized than human natural language. Language in reality is open and variable, hence there are often some general or not succinct expressions, which affect the practical application effect of the VQA model based on the task decomposition. In the NS-VQA method, reasoning execution mainly relies on symbolic reasoning and program calls, that is, pre-set reasoning programs are called during the symbolic reasoning process. As the complexity of reasoning increases, it becomes difficult to pre-set reasoning programs.

Compared with the above methods, our work has the advantage that we pay attention to the implicit structural information in the language in addition to the word semantic information. Compared with serialized data, graph structural data can effectively express and use the information. The task decomposition network proposed in this article is based on the graph-to-sequence architecture. In order to better perform node embedding, we adopt two methods to establish graph structure data from the input: pre-defined graphs and adaptive graphs. The Graph-to-Sequence Task Decomposition Network learned from graph data can better adapt to non-normalized natural languages, improve task decomposition effect, and improve the accuracy of VQA tasks, compared with Seq2Seq networks. In addition, for reasoning execution in complex scenarios, we propose a reasoning executor that gradually executes the reasoning steps through message transmission between networks.

## Methods

For the visual reasoning required in VQA tasks, it is necessary to clarify the goals and steps of the reasoning, obtain relevant visual clues, and through a series of tinny reasoning operations, get the final result. Therefore, our VQA model is based on a task decomposition framework, which guides the model to gradually reason and obtain the answer through task decomposition. We have listed in Table 2 the comparison between the structure of our Graph2Seq-TDN model and the Seq2Seq-based model as well as the Graph-based model. Compared with these two architectures, our Graph2Seq-based model can capture rich structural information while having strong sequence generation capabilities. We have adopted two methods for preprocessing input graph data, one is pre-defined graph and the other is adaptive graph. In addition, we have designed an additional reasoning executor for real-world scenarios to avoid manually defining too many reasoning modules.

**Table 2:** Comparison of Model Structures.

Method	Seq2Seq-based model	Graph-based model	Graph2Seq-TDN
**Instance**	[32], [33,35–39],	[22], [23,24]	–
**Core architecture**	Encoder-decoder based on RNN/LSTM	Graph Neural Networks (GCN, GAT, etc.)	Graph Encoder (GNN variant) + Sequence Decoder (RNN/LSTM)
**Input representation**	Sequence (Text)	Graph structure (nodes, edges)	Graph structure (nodes, edges)
**Preservation of structural information**	Weak	Strong (direct processing of graph structure)	Strong (direct processing of graph structure)
**Sequence generation capability**	Strong (expertise in generating sequences)	Weak (usually used for node/graph classification, non-generative)	Strong (integrated with attention-based decoder, dynamically focusing on different parts of the graph)
**Main advantages**	Mature sequence processing, powerful generation capability	Rich capture of structural information	Balancing structure and sequence generation to handle complex relationships

**Fig 4:**
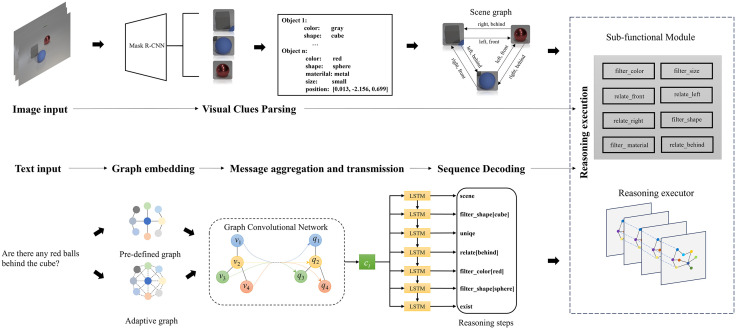
Overview of visual question answering model.

The overall model is shown in Fig 4, including a visual clues parser, a task decomposition network, and the reasoning executor. The visual clues parser is used to extract the visual information required for reasoning, the task decomposition network parsing the entire reasoning task into multiple sub-tasks based on natural language prompts. The model first extracts visual cues from the input image data and organizes them into a structured scene graph. Simultaneously, the input natural language question is embedded into a graph-structured representation and fed into a task decomposition network, where its feature representation is refined through multiple rounds of iterative optimization. Subsequently, the decoder parses the updated features into a sequence of reasoning steps. These steps, along with the visual scene graph, are then input into a reasoning execution module for joint inference, ultimately generating the final answer. The relevant framework and principles will be further introduced in detail below.

## Scene parsing

The acquisition of visual clues is a necessary part of visual reasoning, including the attributes, positions, and relationships between entities in the scene. Organizing information by constructing a scene graph is an effective approach. Our model uses the Mask R-CNN network to perform entity detection, the detection results are then sent to a simple attribute detector, which obtains relevant information about each entity, and ultimately generates a scene graph U = {O_*1*_, O_*2*_, …, O_*n*_}, where *n* is the total number of entities in the scene. For the *i*-th entity O_*i*_, O_*i*_ = {$p_{position}^{i}$, $p_{color}^{i}$, $p_{shape}^{i}$, $p_{material}^{i}$, $p_{size}^{i}$}, where *p^i^_position_*, *p^i^_color_*, *p^i^_shape_*, *p^i^_material_* and *p^i^_size_* respectively represent the position, color, shape, material, and size attributes of the *i*-th entity.

### Graph-to-sequence task decomposition network

Clarifying reasoning steps is a prerequisite for visual reasoning. In the VQA task, we parse the reasoning steps from the natural language question, and thus decomposing the reasoning task. To improve the effectiveness of task decomposition, we propose the Graph-to-Sequence Task Decomposition Network (Graph2Seq-TDN). Compared to serialized data, Graph2Seq-TDN uses graph structural data to represent natural language, which can include syntactic structure information to enrich the data information. Therefore, we use GCN and LSTM to construct a Graph-to-Sequence (Graph2Seq) structure encoder-decoder network for processing graph structural data. The Graph structural data has multiple types, which can generally be divided into pre-defined graph (dependency graph, constituency graph, AMR graph, etc.) and adaptive graph. In this work, we use both dependency graph and adaptive graph to preprocess textual data, and we will provide a detailed introduction to the process of using these two types of graph data for task decomposition.

### Task decomposition with pre-defined graph

**Fig 5:**
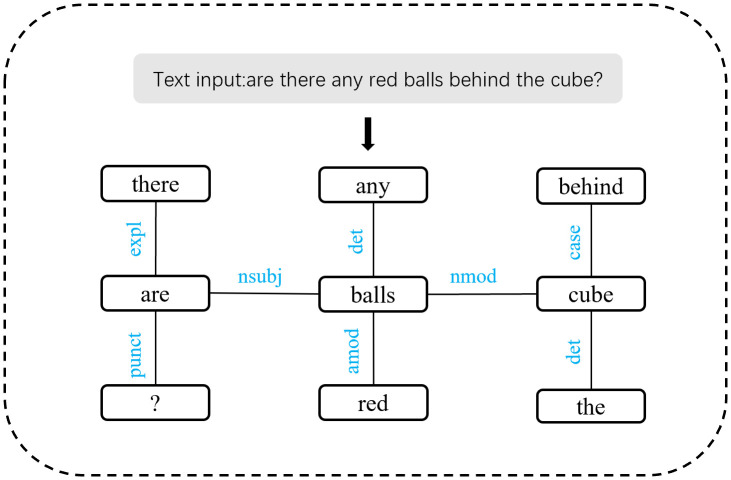
Dependency graph constructed by Stanford NLP.

Building a pre-defined graph refers to building the graph structure during preprocessing by leveraging existing relationship resolution tools or manually defined rules. A dependency graph mainly describes the dependencies between different words in a given sentence, the dependency graph for the question “Are there any red balls behind the cube?”, as parsed by Stanford NLP, is shown in Fig 5.

As we can see, the dependency graph contains multiple edges representing dependencies between words. The entire dependency graph will be used as a heterogeneous pre-defined graph to train the task decomposition network. During this process, the edges carrying syntactic information will play an important role in the transmission and updating of messages. The process is as follows:

**Graph structure data embedding.** For the input graph data *G* = (*V*, *E*), where *V* is the set of nodes and *E* is the set of edges, computing the adjacency matrix *A*_*pre*_ and degree matrix *D*_*pre*_, and embedding the nodes and edges:


$$v_{i}^{*}=W_{N}v_{i},\;e_{ij}^{*}=W_{E}e_{ij}\;$$ (1)

where *v*_*i*_ represents the *i*-th node, v* irepresents the embedded feature vector of the *i*-th node, *e*_*ij*_ represents the edge between node *v*_*i*_ and node *v*_*j*_, e* ij represents the embedded feature vector of edge *e*_*ij*_, *W*_*N*_ and *W*_*E*_ respectively represent the weight matrix for node and edge embedding. Then, the set of embedded node features is V* and the set of embedded edge features is E*.

**Message aggregation and transmission.** Constructing a L-layer GCN with input graph feature data set *G*_*f*_ ={*A*_*pre*_, *D*_*pre*_, ***cat***(V*, E*)}, where ***cat*** represents the concatenation operation, and propagating and updating node and edge information layer by layer:


$$Q^{\left(l+1\right)}=\sigma \left(\Gamma Q^{\left(l\right)}W^{\left(l\right)}+b^{\left(l\right)}\right)$$ (2)

$$\Gamma ={D_{pre}}^{-\frac{\text{1}}{\text{2}}}\left(D_{pre}-A_{pre}\right){D_{pre}}^{-\frac{\text{1}}{\text{2}}}$$ (3)

where *Q*^(*l*)^ represents the feature information of the *l*-th GCN layer, *Q*^(0)^ = ***cat*** (V*, E*).*W*^(*l*)^and *b*^(*l*)^ represent the weight matrix and bias matrix of the *l*-th GCN layer, *σ* denotes the nonlinear activation function, and Γ is the Laplacian matrix.

**Decoding of sequence structured data.** Calculating the context vector *c*_*t*_ at time *t* based on the data feature *Q*^(*L*)^ calculated from the last GCN layer of the encoder and *α*_*i*_:


$$c_{t}=\sum {\mathrm{\backslash nolimits}}_{i}^{n}\alpha_{i}q_{i}^{\left(L\right)}$$ (4)

where *α*_*i*_ is the attention value for the *i*-th feature vector. The attention weight *α*_*i*_ is calculated from the hidden state *h*_*t*_:


$$\alpha_{i}=\frac{\exp\left(h_{t}^{T}W_{A}q_{i}^{\left(L\right)}\right)}{\sum {\mathrm{\backslash nolimits}}_{j=0}^{n}\exp\left(h_{t}^{T}W_{A}q_{j}^{\left(L\right)}\right)}$$ (5)

where, *W*_*A*_ is the attention weight matrix, *q(L) i*i is the *i*-th feature vector in *Q*^(*L*)^, obtaining the hidden state *h*_*t*_ at current time t through the LSTM network on the decoder side:


$$h_{t}=LSTM\left(h_{t-1},y_{t-1}\right)$$ (6)

where *h*_*t-1*_ is the hidden state at previous time *t*-1, and *y*_*t-1*_ is the output token at the previous time *t*-1.

Finally, the context vector,together with the decoder output, is passed to a fully connected layer with softmax activation to obtain the distribution for the predicted token:


$$p\left(y_{t}|h_{t},c_{t}\right)=soft\max\left(h_{t},c_{t}\right)$$ (7)

The overall loss function is as follows:


$$l_{cross}=-\sum {\mathrm{\backslash nolimits}}_{t}^{N}{y^{\prime }}_{t}\log p_{t}$$ (8)

where *y*_*t*_′ is the true label of the *t*-th token, and *p*_*t*_ is the distribution for the *t*-th predicted token.

### Task decomposition with adaptive graph

The advantage of pre-defined graphs lies in encoding prior knowledge of the data into the graph structure, but this prior knowledge requires extensive human efforts and domain expertise, and it is easy to introduce noise when constructing the graph. Therefore, using adaptive graphs is a more efficient approach. The process of constructing adaptive graphs is trained end-to-end along with downstream tasks. The specific process is as follows:

**Graph structure data embedding** For the input graph data *G*, node embeddings are obtained by embedding the nodes *V* according to (1) to get the node feature matrix V*. Due to the use of adaptive graphs, which means that the structure of the graph is not fixed, only node features are embedded and encoded.

**Similarity matrix calculation** Similarity measurement is performed on the nodes in the original graph by using *m* weight vectors *w*_*p*_ (each representing a perspective) to calculate *m* independent similarity matrices with the cosine similarity function. The average of these matrices is then taken as the final similarity results in the similarity matrix S.


$$s_{ij}=\sum {\mathrm{\backslash nolimits}}_{p}^{m}\frac{\left(w_{p}⊙v_{i}\right)\cdot \left(w_{p}⊙v_{j}\right)}{\left\|w_{p}⊙v_{i}\right\|\cdot \left\|w_{p}⊙v_{j}\right\|}$$ (9)

**Message aggregation and transmission** Then extracting a symmetric sparse non-negative adjacency matrix *A* from S. A L-layer GCN network is established, taking the feature matrix V*, adjacency matrix *A*, and degree matrix *D* as input. According to (2) ~ (3), node updates are performed layer by layer, and according to (10) ~ (11), the adjacency matrix *A* is updated and combined with the original adjacency matrix *A*^(0)^.


$$A\prime^{\left(l\right)}=\delta_{1}\Gamma^{\left(0\right)}+\delta_{2}\left\{\gamma_{1}f\left(A^{\left(l\right)}\right)+\gamma_{2}f\left(A^{\left(1\right)}\right)\right\}$$ (10)

$$\Gamma^{\left(0\right)}={\left(D\right)}^{-\frac{\text{1}}{\text{2}}}A^{\left(0\right)}{\left(D\right)}^{-\frac{\text{1}}{\text{2}}}$$ (11)

where *η* and *γ* are combination weight hyperparameters, *A*^(*l*)^is the updated adjacency matrix in *l*-th GCN layer, and *f* denotes row normalization of matrix *A*^(*l*)^.

**Decoding of sequence structured data** According to (4) ~ (7), calculating the context vector *c*_*t*_ and pass it along with the decoder output to a fully connected layer with softmax activation to obtain the distribution of predicted tokens. Calculating graph regularization loss as:


$$l_{G}=\frac{\text{1}}{n^{2}}(\kappa \cdot \sum {\mathrm{\backslash nolimits}}_{i,j}{A_{ij}\left\|q_{i}^{\left(L\right)}-q_{j}^{\left(L\right)}\right\|}^{2}+\eta {\left\|A^{\left(L\right)}\right\|}^{2}+n\mu \log(A^{\left(L\right)}))$$ (12)

where *κ*, *η*, *μ* are non-negative hyperparameters. And the overall loss function is as follows:


$$l_{total}=l_{cross}+l_{G}$$ (13)

### Reasoning execution

#### Compositional reasoning with the sub-functional modules.

**Fig 6:**
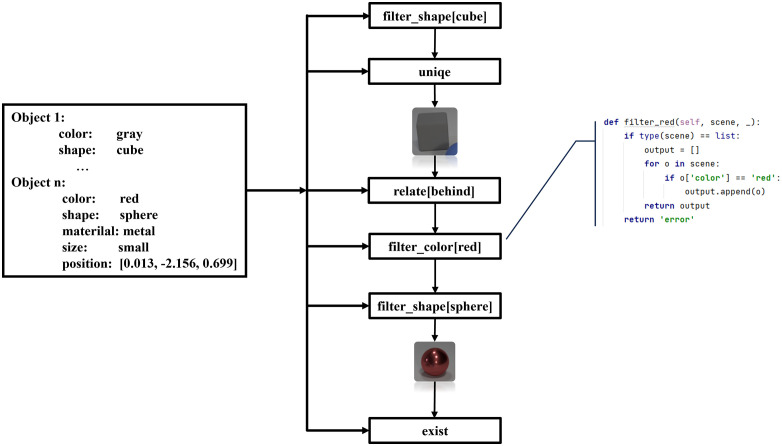
Compositional reasoning with the sub-functional modules.

After obtaining the corresponding visual clues and accurate task decomposition steps, the approach of symbolic reasoning is to obtain the final answer by calling sub-functional modules for combinatorial reasoning, as shown in Fig 6. The process effectively implements formal logic within a computational framework by manipulating symbolic representations of visual entities—such as objects, attributes, and spatial relations—according to predefined logical rules. Each sub-functional module corresponds to a distinct inferential capability, such as attribute verification, relation comparison, or temporal projection, thereby encapsulating a specific logical operation or rule set. The combinatorial reasoning mechanism orchestrates these modules by executing a structured inference chain.

A key advantage of this symbolic architecture is its transparency and interpretability. Unlike opaque neural embeddings, the intermediate outputs of each module—such as newly derived relations or validated causal conditions—are explicitly traceable. This step-by-step derivation mirrors the rigor of formal proof systems in logic. Ultimately, the final answer is generated through a deterministic synthesis of module outputs, ensuring high precision in domains that demand rigorous logical reasoning.

#### Compositional reasoning with reasoning executor.

For scenarios with simple content, calling preset programs for symbolic reasoning can work well to obtain accurate results, because simple scenarios only involve limited object categories, attributes, and spatial relationships, which can be covered in the preset programs. However, in the face of complex scenarios, the original reasoning execution methods are difficult to meet this completeness requirement. Therefore, we propose a reasoning executor to deal with complex scenarios, making the execution of reasoning steps simple and efficient.

**Fig 7:**
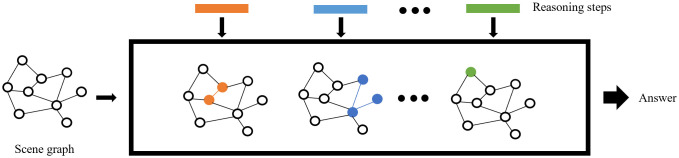
Reasoning executor structure.

Fig 7 shows the structure of the reasoning executor, which is also implemented based on graph neural networks. We have established an M-layer graph neural network, which combines reasoning steps and scene information through information transfer between networks, making reasoning execution concise.

Assuming that the task decomposition steps P = [*p*_1_, *p*_2_, ……, *p*_*n*_] of length *n* are resolved from question Q, and *n* ≤ M, the scene graph U is represented as U = (V_*s*_, E_*s*_), where V_*s*_, E_*s*_ represents the point set and edge set. After embedding nodes and edges, the scene graph U and reasoning step P are fed into the reasoning executor. The reasoning step information is transmitted and updated in the M-layer network with the node and edge information:


$$q_{i}^{M}=v_{j}^{M-1}⊗p_{M}$$ (14)

$$e_{ij}^{M}=e_{ij}^{M-1}⊗p_{M}$$ (15)

The information transmission from node *i* to *j* in the M-layer is as follows:


$$v_{i}^{M}=r\left(\sum_{j}\alpha_{ij}^{M}W^{M}q_{i}^{M}\right)$$ (16)

where, *r* represents the activation function, *W*^*M*^ represents the corresponding learnable weight, and $\alpha_{ij}^{M}$ represents the attention value between two nodes, calculated through the eigenvalues of nodes *i*, *j*, and edges *e*_*ij*_:


$$\alpha_{ij}^{M}=\frac{f\left(q_{i}^{M},q_{j}^{M},e_{ij}^{M}\right)}{\sum_{j}^{N_{i}}f\left(q_{i}^{M},q_{j}^{M},e_{ij}^{M}\right)}$$ (17)

where *f* (·) represents multi-layer perceptron, and N_*i*_ represents all nodes connected to node *i*.

### Experiment

To validate the effectiveness of the Graph2Seq-TDN proposed in this work for solving VQA tasks, experiments were conducted on CLEVR family (including CLEVR, CLEVR-CoGenT and CLEVR-Humans) and GQA, VQA v2 datasets, and it was compared with baseline models to evaluate the performance. The experimental settings and result analysis will be described in detail below.

### Experiment setting

#### Datasets.

We tested the performance of the model on multiple datasets. CLEVR is a standard synthetic dataset used to test the model’s combinatorial reasoning ability. CLEVR-CoGenT and CLEVR-Humans are derivative datasets of CLEVR, both of which pose generalization challenges to the model. The GQA dataset comes from real-world scenarios and covers a wider range of issues. The specific introductions of each dataset are as follows:

**CLEVR** Compositional Language and Elementary Visual Reasoning (CLEVR) dataset is a large synthetic dataset that contains 100000 visual images consisting of simple geometric objects and 1000000 natural language question-answer pairs which from 90 question families and involving complex reasoning. It can be seen that the advantage of CLEVR dataset compared to previous VQA dataset is the reduction of dataset bias. In order to focus on visual reasoning ability, the complexity of the scene is simplified while increasing the difficulty of the questions. The main types of questions in volve querying attribute, comparing attributes, existence, counting and integer comparison.

**CLEVR-CoGenT** The CLEVR-CoGenT dataset is derived from CLEVR and the dataset was used to study the ability of models to recognize novel combinations of attributes at test-time. The data is generated in two different conditions: in condition A, cubes are gray, blue, brown, or yellow, cylinders are red, green, purple, or cyan, and spheres can have any color; in condition B, cubes are red, green, purple, or cyan, cylinders are gray, blue, brown, or yellow, and spheres can have any color.

**CLEVR-Humans** The dataset was written by workers on Amazon Mechanical Turk according to CLEVR images, different from CLEVR dataset, CLEVR-Humans dataset has 32164 natural language question-answer pairs which exhibit more linguistic variety than synthetic CLEVR questions, and hence be more challenging.

**GQA [44]** The dataset is designed for real-world visual reasoning and combinatorial question answering, drawing inspiration from the CLEVR task and comprising 113000 images from COCO and Flickr and 22 million distinct questions, each image is annotated with a dense Scene Graph, representing the objects, attributes and relations it contains. Each question is associated with a functional program which lists the series of reasoning steps needed to be performed to arrive at the answer. Each answer is augmented with both textual and visual justifications, pointing to the relevant region within the image. GQA questions tend to involve more elements from the image compared to VQA questions, and are longer and more compositional as well. Conversely, VQA questions tend to be a bit more ambiguous and subjective, at times with no clear and conclusive answer. GQA provides more questions for each image and thus covers it more thoroughly than VQA.

**VQA v2** The VQA v2 dataset is a large-scale, human-annotated benchmark for visual question answering tasks, designed to reduce language bias by pairing each question with multiple images that yield different answers, thus emphasizing the importance of image understanding. It contains over 265,000 images sourced primarily from the MS COCO dataset, accompanied by approximately 1.1 million questions and 10 million answers. Each question has 10 human-annotated answers to ensure robustness, and the dataset requires models to integrate computer vision, natural language processing, and commonsense reasoning to generate accurate responses. It serves as a foundational resource for evaluating multimodal AI systems.

### Experimental environment setup

The training process of the model is divided into two stages: image detection and task decomposition. We use Mask R-CNN network to process images and train it on four NVIDIA Tesla K80 GPUs. The experimental environment is Linux Ubuntu 16.04, Python 3.6, CUDA 9.0, PyTorch 0.4.1; at the task decomposition stage, the model was trained on one NVIDIA GeForce RTX 2080 GPU in a Linux Ubuntu 18.04 experimental environment, Python 3.8, CUDA 11.0, PyTorch 1.7.1.

### Evaluation metrics

**Overall answering accuracy** Accuracy of answering questions.

**Program accuracy** It represents task decomposition accuracy, before providing the correct answer to a question, it is necessary to break down the task into steps, which manifest as an executable program sequence, this metric is used to evaluate the accuracy of the overall generated program sequence, it measures the model’s ability of combine reasoning.

**BLEU** In addition to accuracy metrics, we also used the BLEU metric to evaluate the performance of our models. BLEU measures the similarity between two sequences, and it can be divided into four common indicators: BLEU-1, BLEU-2, BLEU-3, and BLEU-4 based on n-gram, where n represents the number of consecutive elements in the sequence. We divided the generated sequence and standard sequence into multiple sub-sequence sets according to n-gram, and then investigated how many corresponding subsequences in these two sets. We used BLEU to evaluate the models’ ability to select reasoning modules.

#### Model comparison.

In Table 3, we list the characteristics of the comparative models to facilitate the evaluation of the true advantages of the proposed method in conjunction with experimental performance. Focusing on the three dimensions of computational cost, interpretability, and robustness, we compared and analyzed these models by incorporating their design features and general knowledge in the field of machine learning, with the summaries provided in Table 4.

**Table 3:** Model Characteristics and Performance Comparison.

Model Name	End-to-End	Task-Decomposition	Pre-training	Fine-tuning	Explicit Reasoning
**Mac**	No	Yes	No	No	No
**NS-VQA**	No	Yes	No	No	Yes
**NMN**	No	Yes	No	No	Yes
**NS-ASP**	No	Yes	No	No	Yes
**TbD-net**	No	Yes	No	No	Yes
**DMN**	No	Yes	No	No	Yes
**NS-CL**	No	Yes	No	No	Yes
**LR-Capsule**	Yes	No	No	No	No
**FiLM**	Yes	No	No	No	No
**BLIP**	Yes	No	Yes	Yes	No
**InstructBLIP**	Yes	No	Yes	Yes	No
**Cola**	Yes	No	Yes	Yes	No
**Graph2Seq-TDN**	No	Yes	No	No	Yes

**Table 4:** Model Attributes Overview.

Model Type	Computational Cost	Interpretability	Robustness	Representative Models
**Task Decomposition + Explicit Reasoning**	Medium (Multi-module, Multi-step reasoning)	Very High (Transparent process, Easy to trace)	Medium (Depends on module stability, but easy to target improvements)	NS-VQA, NMN, TbD-net, DMN, Graph2Seq-TDN
**Task Decomposition (No Explicit Reasoning)**	Medium (Multi-module)	High (Modular design)	Medium	Mac
**End-to-end + Pre-training + Fine-tuning**	Very High (Pre-training is extremely costly)	Low (Black-box model)	High (Benefits from large-scale pre-training)	BLIP, InstructBLIP, Cola
**End-to-end (No Pre-training)**	Low to Medium (Fewer parameters, no pre-training overhead)	Low (Black-box model)	Medium (Depends on task-specific training data)	LR-Capsule, FiLM

End-to-end models (e.g., FiLM, BLIP, InstructBLIP, Cola) typically incur high computational costs. They learn direct mappings from input to output through a single, complex model, often characterized by a large number of parameters, and require substantial data and computational resources for pre-training and fine-tuning. Their advantage, however, lies in the potential for achieving higher performance through joint optimization. Task-decomposition models (e.g., NS-VQA, NMN, TbD-net, DMN, Graph2Seq-TDN) typically break down complex tasks into sub-modules or sub-problems. The computational cost of this design varies with implementation; on one hand, specific sub-modules can be optimized, but on the other hand, the series connection of multiple modules may lead to error accumulation, and the design and training of the overall system can be equally complex. Models that undergo pre-training and fine-tuning stages (e.g., BLIP, InstructBLIP, Cola) usually have very high total computational costs. Pre-training, especially on large-scale datasets, consumes enormous resources. Although fine-tuning is less costly, it still requires additional computation. For models that do not use pre-training, their computational cost is primarily concentrated on training on specific task datasets, which may be relatively lower, but their performance ceiling might also be limited as a result.

Almost all models employing a task-decomposition architecture (e.g., Mac, NS-VQA, NMN, NS-ASP, TbD-net, DMN, NS-CL, Graph2Seq-TDN) possess inherent advantages in interpretability. Their modular design allows researchers and developers to trace intermediate results, understand the functionality of each sub-module, and diagnose the location of errors. Models with explicit reasoning capabilities (e.g., NS-VQA, NMN, NS-ASP, TbD-net, DMN, NS-CL, Graph2Seq-TDN) are typically more interpretable, as they can generate intermediate reasoning steps similar to a “chain of thought,” making their decision-making process more transparent, understandable, and trustworthy for humans. In contrast, end-to-end models (e.g., LR-Capsule, FiLM, BLIP, InstructBLIP, Cola) are often regarded as “black boxes,” with internal decision-making processes that are difficult to interpret directly. Although they may deliver powerful performance, understanding “why” a particular decision is made remains challenging, often requiring reliance on post-hoc interpretation techniques to enhance their explainability.

The robustness of task-decomposition models depends on the stability of their sub-modules. The failure of a single sub-module could trigger a cascade of errors. However, this design also allows for independent improvement and enhancement of vulnerable sub-modules, potentially boosting the overall system’s robustness. The robustness of end-to-end models is built upon the holistic optimization of their parameters. A well-trained and data-sufficient end-to-end model may demonstrate strong tolerance to certain types of noise. However, its vulnerabilities can also be difficult to locate and rectify. Models that undergo large-scale pre-training (e.g., BLIP, InstructBLIP, Cola) generally exhibit stronger generalization capabilities and robustness. This is because they learn richer and more stable feature representations from massive datasets, making them less sensitive to minor perturbations in these features. Models equipped with explicit reasoning abilities, if their reasoning mechanisms are well-designed, may generalize better to unseen data or scenarios beyond their training distribution, as they rely on logic and rules rather than merely statistical correlations. This contributes to enhancing their robustness in new environments.

In summary, end-to-end models (e.g., BLIP, InstructBLIP) typically incur high computational costs due to pre-training and fine-tuning processes and exhibit poorer interpretability owing to their “black-box” nature. However, they may achieve certain robustness advantages through large-scale pre-training. In contrast, task-decomposition architecture models (e.g., NS-VQA, NMN) enhance interpretability and debugging convenience via modular design. Their computational cost and robustness highly depend on the specific implementation and stability of sub-modules. For instance, models with explicit reasoning capabilities may increase computational overhead due to multi-step reasoning but can also contribute to improved generalization robustness. Overall, end-to-end models may leverage scale effects to achieve performance advantages when data is sufficient, while task-decomposition models excel in process transparency and controllability. The choice of model requires a trade-off based on practical needs among efficiency, transparency, and stability.

### Evaluation and analysis on experiment results of answering accuracy

Firstly, experiments were conducted on the CLEVR dataset. As a large dataset, CLEVR contains a variety of question types and is suitable for evaluating a model’s reasoning abilities. We randomly selected 10 questions from each question family in the CLEVR dataset to obtain a dataset with a total of 900 program annotations for training, and performed a test on the test set. The experimental results are shown in Table 5.

**Table 5:** CLEVR accuracy (overall and per-question-type) by compared methods and Graph2Seq-TDN.

Method	Program Annotations	Answer Annotations	Count	Compare Numbers	Exist	Query Attribute	Compare Attribute	Overall
**Human**	–	–	86.7	86.4	96.6	95.0	96.0	92.6
**SAN [45]**	700k	–	52.2	73.5	71.1	85.3	52.3	68.5
**DMN [46]**	–	–	66.0	67.2	87.4	–	67.2	86.9
**PG + EE [35]**	9k	0	79.7	79.1	89.7	92.6	96.0	88.6
**PG + EE**	700k	0	92.7	98.7	97.1	98.1	98.9	96.9
**TbD-net [47]**	700k	0	97.6	99.4	99.2	99.5	99.6	99.1
**FiLM [48]**	0	700k	94.3	96.8	99.1	99.1	99.1	97.7
**MAC [49]**	0	700k	97.2	99.4	99.5	99.3	99.5	98.9
**NS-CL [50]**	0	700k	98.2	99.0	98.8	99.3	99.1	98.9
**NS-VQA [39]**	270	700k	99.7	99.9	99.9	99.8	99.8	99.8
**Graph2Seq-TDN (pre-defined)**	900	0	99.7	96.1	99.5	99.8	99.8	99.5
**Graph2Seq-TDN (adaptive)**	900	0	99.9	99.7	99.7	99.9	99.9	99.9

In Table 5, we present detailed experimental result information for our proposed model and compared models, including the number of program annotations and answer annotations used in training, accuracy in answering overall questions, and accuracy in answering different types of questions. Through the information, we not only compared the overall accuracy of different models in answering questions, but also evaluated their ability to answer different types of questions, as well as the training costs of different models in achieving similar accuracy. Overall, our model achieved a high accuracy of 99.9% with adaptive graph and 99.5% with pre-defined graph in answering overall questions, and achieved over 99% accuracy for all question types with adaptive graph, and for all question types except ‘compare number’ with pre-defined graph. Among all compared methods, TbD-net and NS-VQA achieved the closest overall accuracy with 99.1% and 99.8% respectively. In terms of training cost, TbD-net used 700k program annotations for training without using any answer annotations, and NS-VQA used 270 program annotations for pre-training and further trained on 700k answer annotations for reinforcement training, while Graph2Seq-TDN used only 900 program annotations for training without any answer annotations.

We have listed the question answering accuracy of the model on different datasets in Table 6 and 7, and compared it with recent methods. Table 6 shows the experimental results of the model on the CLEVR dataset family. The results on the CLEVR dataset show that Graph2Seq-TDN has the highest accuracy in answering questions. Among them, Cola and InstructBLIP are based on large language models and multimodal large models, and their results were fine-tuned through one epoch on the CLEVR dataset. It can be seen that both two models have shortcomings in visual reasoning and are not as effective as specialized models for handling visual reasoning tasks.

**Table 6:** Question answering accuracy (overall) on CLEVR family benchmarks.

Benchmark	Method	Overall
**CLEVR**	InstructBLIP [29]	33.7
	Cola [31]	54.3
	NMN [32]	73.2
	Tensor-NMN [51]	96.4
	NS-ASP [40]	96.71
	State-Input Transformer [52]	96.8
	Transformer	97.4
	TMN [38]	98.0
	Graph2Seq-TDN	99.9
**CLEVR-HUMAN**	PG + EE	66.6
	NS-VQA	67.0
	Graph2Seq-TDN (pre-defined)	70.3
	Graph2Seq-TDN (adaptive)	71.7
**CLEVR-CoGenT**	MAC	96.9A/79.5B
	Transformer	97.5A/78.9B
	TMN	97.9A/80.6B
	NS-CL	97.9A/74.1B
	VSACL [53]	98.0A/91.9B
	LR-Capsule [54]	98.1A/85.6B
	FiLM	98.3A/78.8B
	TbD	98.8A/75.4B
	NS-VQA	99.8A/63.9B
	Graph2Seq-TDN (pre-defined)	99.2A/92.4B
	Graph2Seq-TDN (adaptive)	99.8A/94.7B

**Table 7:** Question answering accuracy (overall) on real scene benchmark.

Benchmark	Method	Overall
**GQA**	BLIP [28]	41.7
	InstructBLIP	49.2
	Cola	60.3
	NS-VQA	65.0GT/53.2DET
	Graph2Seq-TDN (pre-defined)	68.8GT/56.5DET
	Graph2Seq-TDN (adaptive)	70.4GT/57.9DET
**VQA v2**	NMN	57.3
	DMN	66.8
	Cola	83.7
	Graph2Seq-TDN	65.9

To further validate the model’s adaptability to natural language and reasoning ability, we conducted experiments on the CLEVR-Human dataset. We using the results from training on the CLEVR dataset and fine-tuned our model on 600 manually annotated programs of CLEVR-Human dataset, and added statistical modules to the basic sub-functional modules. Table 6 shows the comparative results of the experiments. It can be seen that compared to the near 100% accuracy on the CLEVR dataset, both our model and the compared model have declined in accuracy on the CLEVR-Human dataset. However, our model still achieves a higher accuracy than the compared model by 4.7%, it indicates that Graph2Seq-TDN has better natural language adaptability than the compared model.

We tested the model on the CLEVR-CoGenT dataset to evaluate generalization ability on unseen attribute combinations, we trained the model on training set of split A and evaluated it on validation sets of split A and split B respectively. It can be seen that due to the presence of unseen attribute combinations in split B, the accuracy of all models trained on split A has decreased to a certain extent on split B. Graph2Seq-TDN achieved good performance on both split A and split B, indicating that the model has good generalization ability on the dataset and reduces its dependence on data bias during questions answering.

We also validated it on real-world datasets. Compared with the simple geometric scenes in CLEVR, the real scenes in GQA and VQA v2 are more complex, with richer types, attributes, and relationships of items. The GQA dataset emphasizes combinatorial reasoning and provides specific task decomposition annotations, while VQA v2 is a more general benchmark for visual question answering tasks. We trained and validated on GQA’s balanced data that has smaller dataset bias. We used both the self-contained scene graph in the dataset and the detected scene graph, and the experimental results are shown in Table 7. ‘GT’ represents that we employed the ground truth scene graphs which come from the annotation provided by the dataset, and ‘DET’ represents the scene graphs come from detection.

The experimental results on the GQA dataset show that unlike the evaluation results on benchmarks of CLEVR family, the models’ answering accuracy on the GQA dataset are generally around 60%, the answering accuracy of Graph2Seq-TDN is about 70% when using the ground truth scene graph, while the answering accuracy decreased to about 55% when using the detected scene graph, this indicates that accurate acquisition of visual information is crucial in the process of visual reasoning, compared to the simple scenes in the CLEVR dataset, the complex real-world scenes in GQA pose significant challenges for extracting visual information. Our model achieved an accuracy of 65.9% on the VQA v2 dataset, performing comparably to DMN but lower than the Cola model; however, on benchmarks requiring compositional reasoning such as GQA and CLEVR, where the Cola model achieved accuracies of 60.3% and 54.3% respectively, our model significantly outperformed Cola, highlighting its strengths in structured reasoning tasks. This advantage enables it to extend to open-world scenarios; however, in real-world applications, the model’s performance is highly dependent on high-quality scene graph inputs, as the accuracy of scene graphs directly affects object detection and relationship recognition precision—for instance, the model needs to accurately identify spatial relationships such as “a table next to a chair” or “a person sitting on a chair.” If the scene graph parser contains errors or omits critical relationships, the model’s reasoning chain may break, leading to performance degradation. Additionally, while the model demonstrates adaptability to diverse linguistic structures, this adaptability typically requires fine-tuning for specific languages or domains; otherwise, its generalization potential may not be fully realized in unknown scenarios. In summary, our model excels in compositional reasoning and linguistic generalization but still requires enhanced robustness in visual information extraction and optimized fine-tuning strategies to further improve its applicability in real-world settings.

### Evaluation and analysis on experiment results of task decomposition

We hope that the model can obtain answers according to human logic, so in addition to question answering accuracy, we also need to pay attention to the accuracy of task decomposition, including two indicators: Program accuracy and BLUE, we evaluated the model’s task decomposition ability on three datasets: CLEVR, CLEVR-CoGenT, and GQA (CLEVR-Human lacks relevant annotations), the relevant results are shown in Table 8.

**Table 8:** Experiment results of task decomposition.

Benchmark	Method	BLEU-1	BLEU-2	BLEU-3	BLEU-4	Program Accuracy
**CLEVR**	**NS-VQA**	0.828	0.807	0.780	0.765	63.2
	**Graph2Seq-TDN (pre-defined)**	0.999	0.999	0.998	0.998	99.2
	**Graph2Seq-TDN (adaptive)**	0.998	0.998	0.997	0.996	99.8
**CLEVR-CoGenT**	**NS-VQA**	0.996A/0.963B	0.984A/0.952B	0.960A/0.947B	0.953A/0.928B	98.9A/89.7B
	**Graph2Seq-TDN (pre-defined)**	0.999A/0.993B	0.983A/0.981B	0.962A/0.958B	0.954A/0.944B	99.2A/91.2B
	**Graph2Seq-TDN (adaptive)**	0.998A/0.985B	0.987A/0.974B	0.976A/0.970B	0.965A/0.953B	99.6A/94.5B
**GQA**	**NS-VQA**	0.984	0.971	0.959	0.946	80.2
	**Graph2Seq-TDN (pre-defined)**	0.987	0.975	0.964	0.952	82.1
	**Graph2Seq-TDN (adaptive)**	0.990	0.979	0.969	0.958	84.0

Table 8 shows the evaluation results of the NS-VQA and Graph2Seq-TDN models under BLEU-1, BLEU-2, BLEU-3, and BLEU-4 metrics, respectively. As the n-gram increases, the scores decrease due to the influence of the reasoning order. However, the Graph2Seq-TDN consistently scores higher than NS-VQA, indicating that the Graph2Seq-TDN is superior in selecting the corresponding reasoning modules, moreover, it can be seen that although Graph2Seq-TDN with pre-defined graphs scored lower in answer accuracy than Graph2Seq-TDN with adaptive graphs, there are cases where its BLEU score is higher than that of the Graph2Seq-TDN with adaptive graph, indicating that the ability in selecting corresponding reasoning modules of using pre-defined and adaptive graphs is equal. The visual reasoning ability of task decomposition VQA method mainly depends on the selection and execution order of corresponding reasoning modules. Considering the results in Table 5, Table 6 and Table 7, the program accuracy of Graph2Seq-TDN with adaptive graph is higher than that of Graph2Seq-TDN with pre-defined graph, indicating that the adaptive graph may be superior to the pre-defined graph in the execution order.

As for predicting programs, NS-VQA had an accuracy of 63.2% on CLEVR, while Graph2Seq-TDN had an accuracy of 99.8%. Comparing with other methods, our proposed method has the advantage of lower training cost with the same overall accuracy in answering questions, and has 36.6% higher accuracy in predicting programs, indicating that the Graph2Seq-TDN model provides correct answers according to our expected logic. The results on the CLEVR-CoGenT dataset show that the program accuracy of Graph2Seq-TDN has decreased compared to split A on split B, but still remains above 90%. The results for the GQA dataset show that Graph2Seq-TDN has the highest program accuracy, up to 84.0%, which is 3.8% higher than NS-VQA. Like the accuracy of answering questions, the performance on GQA has declined compared to the program accuracy of over 90% on the CLEVR dataset, mainly due to the large number of questions and linguistic diversity in the GQA dataset, however the program accuracy still maintains above 80%, this indicates that the model still follows human logic for visual reasoning when answering questions. Furthermore, considering the answering question accuracy of the previous experimental results in Table 6 and 7, compared with the program accuracy, the answering question accuracy has decreased significantly, this indicates that in the entire VQA system, it is not enough to only ensure the correct steps of answering the question. Answering the question needs to combine visual information. If the scene information cannot be well mined, useful visual information cannot be obtained, and the question cannot be answered correctly.

In a word, the task decomposition VQA method is designed to select and execute reasoning modules, and considering the results of both tables, we can conclude that the Graph2Seq-TDN model is more efficient in selecting and executing the corresponding reasoning modules than NS-VQA.

**Fig 8:**
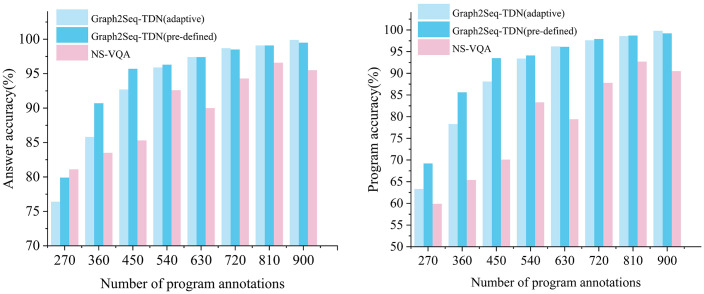
Quantitative results with NS-VQA and Graph2Seq-TDN. (a) Answer accuracy with different number of program annotations;(b) Program accuracy with different number of program annotations.

In addition to the evaluations mentioned above, we conducted a quantitative analysis on CLEVR to further analyze how the accuracy of the predicted programs and final answers varied with the number of program annotation. Results are shown in Fig 8. For answering accuracy in Fig 8a NS-VQA outperforms our model when given 270 program annotations. However, as the number of program annotations increase to 360, the accuracy of Graph2Seq-TDN exceeds that of NS-VQA, and when using 450 program annotations, the difference between the two models is largest that Graph2Seq-TDN with pre-defined graph outperforming NS-VQA by 10.4%. For program accuracy in Fig 8b, Graph2Seq-TDN has always performed better than NS-VQA. It can be seen that as the number of program annotations increased, the program accuracy of NS-VQA gradually improved compared to the results in Table 5 which using supervised training on 270 program annotations and using reinforced training on 700k answer annotations. Additionally, reinforced training improved program accuracy by 3.3%, but its effectiveness is lower than the 18.7% increase that can be seen in answer accuracy.

Furthermore, we can also see that the use of pre-defined graph had better performance when using fewer program annotations, and the use of adaptive graph is more effective when the number of annotations exceeded 630. The results of the quantitative analysis demonstrate that the Graph2Seq-TDN model has better learning ability on the data. Reinforced training can significantly improve answer accuracy but has a lower effect on program accuracy, and when we want the model to obtain answers in a way consistent with human logic, the Graph2Seq-TDN model is a better choice.

### Evaluation and analysis on qualitative results

**Fig 9:**
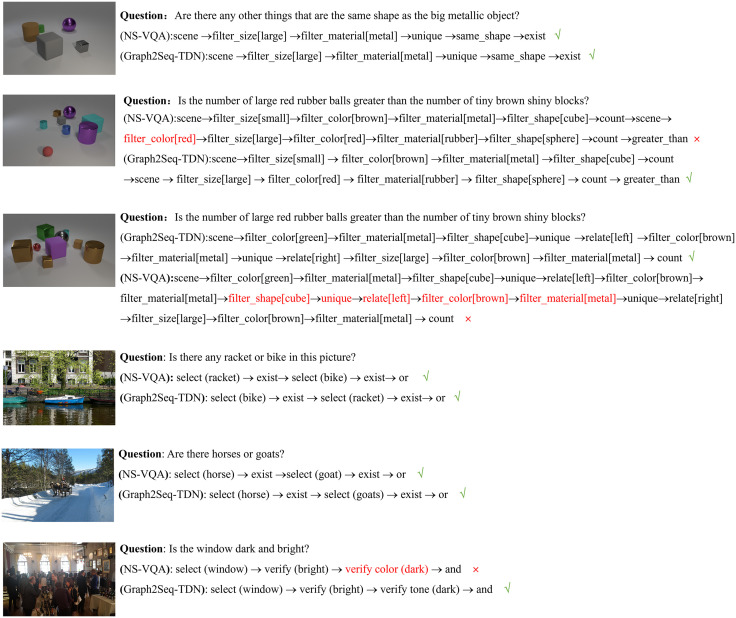
Qualitative results of VQA on CLEVR and GQA.

At the same time, in order to clarify the model’s reasoning process in answering questions, we presented qualitative experimental results as shown in Fig 9, and compared the results of NS-VQA and Graph2Seq-TDN. We illustrated the specific reasoning process of the model in answering questions. For simple questions that do not require executing long program sequences, both NS-VQA and Graph2Seq-TDN models can accurately answer the questions while ensuring that the predicted program matches the program annotation (Fig 9a, Fig 9d, Fig 9e). However, for complex questions with long program sequences, the NS-VQA model may sometimes produce correct answers that do not match the program annotation (Fig 9b). In Fig 9b, the NS-VQA model predicted one extra program module compared with the correct program annotation, and in Fig 9c, the NS-VQA model predicted five extra program modules, which repeated previous reasoning steps. Therefore, it can be seen that when the number of reasoning steps increases, the model may have problems in selecting the program execution order, while Graph2Seq-TDN can still maintain the correct reasoning order for long-step reasoning problems, and Graph2Seq-TDN can still generate appropriate steps for more open and common-sense type of questions in GQA (Fig 9f).

**Fig 10:**
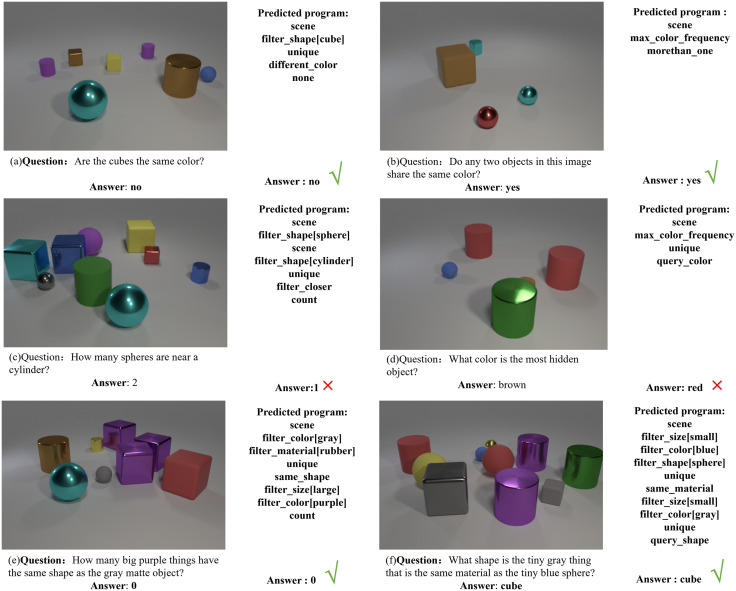
Qualitative results of VQA on CLEVR-Human and CLEVR-CoGenT.

We also present the qualitative experimental results on CLEVR-Human and CLEVR-CoGenT in Fig 10. From the qualitative results, it can be seen that the reasoning problems in the CLEVR-Human dataset are more complex than those in the CLEVR dataset. Although the addition of some reasoning modules can solve some of these problems, they still cannot cover all types of reasoning, such as relationships that are not easily determined like ‘near’ in Fig 10(c) or reasoning types that cannot be solved like ‘most hidden’ in Fig 10(d).

### Ablation analysis

To validate the effectiveness of the proposed Graph2Seq-TDN for visual question answering, experiments were conducted on the GQA dataset to clearly understand how different experimental settings affect the model’s performance in completing visual question answering tasks. The ablated components include the Graph-to-Sequence-based task parser and the GNN-based reasoning executor. In Table 9, the column title ‘Seq2Seq’ indicates the use of a Sequence-to-Sequence-structured LSTM network in the task decomposition stage, while the column title ‘Answer-set programming’ refers to the use of a manually defined logic module. The ablation study demonstrates that the GNN-based configuration significantly improves performance compared to Answer-set programming (an average improvement of approximately 12.2%), validating the effectiveness of the GNN-based reasoning executor. Meanwhile, the Graph2Seq architecture outperforms Seq2Seq in both configurations, proving that the graph structure further optimizes the model’s task decomposition performance. Specifically, the model equipped with the Graph-to-Sequence-based task parser and the GNN-based reasoning executor achieves the best performance (70.4%), indicating that these two components are the most effective designs in this study.

**Table 9:** Performance comparison of model with different components.

Configuration	Answer-set programming	GNN-based
**Seq2Seq**	55.5	67.9
**Graph2Seq**	58.3	70.4

## Conclusion

This work solving the visual question answering task with a task decomposition framework. We introduce the Graph-to-Sequence Task Decomposition Network, which utilizes natural language syntactic structure information through graph-structured data to improve task decomposition performance and thus improve VQA accuracy, and propose reasoning executor for complex scenarios to improve the execution efficiency of reasoning steps. Experimental results on the CLEVR dataset show that our model has better data learning capabilities, requiring less training cost compared to other models to achieve the same accuracy, and achieving high accuracy in both answer and program prediction. Results on the CLEVR-Human dataset show that Graph2Seq-TDN has a better adaptation of the variability of natural language than compared models. The results on the GQA dataset indicate that the VQA system based on Graph2Seq-TDN is better at generating intermediate inference steps for answering questions. However, answering a question requires reasoning steps, relevant inference information, and execution ability. When the obtained information is incomplete or incorrect, the performance of the entire system will be affected. Therefore, how to obtain more effective information in complex scenarios is the subsequent step to be considered.

What’s more, through the above research, it is found that the reasoning models using a task decomposition framework can improve the machine’s ability to handle complex reasoning tasks by parsing natural language to decompose tasks. This framework can be extended to the field of robotics, as robot tasks are essentially highly integrated from basic tasks. For instance, executing high-level tasks like table cleaning requires iterative invocation of primitive functions—’pick up’ and ‘move’ to take objects stay off the table one by one, and re-evaluate the corresponding dynamic scenes in the process. It is also an important extension direction for the future of this research – based on combinatorial reasoning methods to enable robots to adapt to the environment and call basic functions to complete complex tasks. How to improve the model’s combinatorial generalization ability to enable robots to adapt to dynamic environments is the key issue. It can be considered to further expand on sub-functional modules, encapsulate and call existing models to improve their functionality and enable them to interact with the environment, while also dividing tasks more hierarchically. The most important aspect of a task decomposition framework, beyond task decomposition itself, is to use natural language to guide models to solve problems based on human logic, necessitating the transformation of cognitive processes into granular data annotations for training neural networks.

## Supporting information

S1 Data The minimum dataset we provide includes experimental records, original chart files generated from experimental data, and part of processed benchmark data.
(ZIP)
